# *N*-Nitrosodimethylamine Contamination in the Metformin Finished Products

**DOI:** 10.3390/molecules25225304

**Published:** 2020-11-13

**Authors:** Adam Zmysłowski, Iza Książek, Arkadiusz Szterk

**Affiliations:** National Medicines Institute, 30/34 Chełmska, 00-725 Warsaw, Poland; i.ksiazek@nil.gov.pl (I.K.); a.szterk@nil.gov.pl (A.S.)

**Keywords:** NDMA, nitrosamines, GC-MS, metformin

## Abstract

A GC–MS/MS method with EI ionization was developed and validated to detect and quantify *N*-nitrosodimethylamine (NDMA) and seven other nitrosamines in 105 samples of metformin tablets from 13 different manufactures. Good linearity for each compound was demonstrated over the calibration range of 0.5–9.5 ng/mL. The assay for all substances was accurate and precise. NDMA was not detected in the acquired active pharmaceutical ingredient (API); however, NDMA was detected in 64 (85.3%) and 22 (91.7%) of the finished product and prolonged finished product samples, respectively. European Medicines Agency recommends the maximum allowed limit of 0.032 ppm in the metformin products. Hence, 28 finished products and 7 pronged dosage products were found to exceed the acceptable limit of daily intake of NDMA contamination. The implications of our findings for the testing of pharmaceutical products are discussed.

## 1. Introduction

In general, N-nitrosamines (NAs) are the products of reactions between a nitrosating agent and a secondary or tertiary amine; NAs are formed preferentially at elevated temperature. Thus, NAs are mainly detected in food and drinks after processing [1]. In foods, nitrous anhydride is the main nitrosating agent formed from nitrite in an acidic aqueous solution. In drinking water, *N*-nitrosodimethylamine (NDMA) is the most simple and volatile NA that can form during the degradation of dimethylhydrazine (a component of rocket fuel) by chloramination of amine-based precursors or as a byproduct of anion exchange purification of water [2]. NDMA has been shown to be formed in certain foods due to a direct-fire drying process. International Agency for Research on Cancer (IARC) has classified NDMA as a probable carcinogen in humans. NDMA is known to be genotoxic in vivo and in vitro [3,4]. Several case–control studies and a single cohort study of NDMA in humans supported the assumption that NDMA consumption is positively associated with either gastric or colorectal cancer [5,6,7]. Therefore, due to possible contamination of water with NDMA, the World Health Organization (WHO) and U.S. Environmental Protection Agency (EPA) have set the drinking water guideline limits to 100 ng/L and 0.4 ng/L in tap water, respectively [8,9]. Only in a few foods and countries, limits have been set for NAs. In the United States, a limit of 10 µg/kg has been set for total volatile NAs in cured meat products. In 2005, China introduced a limit of 4 and 7 µg/kg of NDMA in fish and related products, respectively [10]. There are currently no maximum regulatory limits for the level of N-nitroso-compounds in food in the European Union [11].

Recently, NDMA and other NAs, e.g., *N*-nitrosodiethylamine (NDEA), have been detected in several pharmaceuticals, including sartans (valsartan, losartan, and candesartan) and raniditine [12,13]. The detection of the NDMA impurity was incidental and happened while performing other tests [14]. Improvement in the analytical techniques enabled the detection of NAs concentrations as low as 1 ng/L [15]. After a thorough investigation, it was discovered that changes in the production process of the active pharmaceutical ingredient (API) are one of the actual reasons for NDMA contamination of valsartan. Due to NDMA contamination, 22 countries issued recalls involving 2,300 valsartan batches from Germany, Norway, Finland, Sweden, Hungary, the Netherlands, Austria, Ireland, Bulgaria, Italy, Spain, Portugal, Belgium, France, Poland, Croatia, Lithuania, Greece, Canada, Bosnia and Herzegovina, Bahrain and Malta [16]. However, in addition to sartans and ranitidine, the European Medicines Agency (EMA) has recently announced that metformin-containing finished products can be contaminated with NDMA [17].

Metformin is the most widely used medication around the world for the treatment of type 2 diabetes mellitus [18]. Usually, metformin is the first-line treatment that works by reducing the production of glucose in the body and reducing glucose absorption from the gut. Metformin is also effective against various conditions, including prediabetes, polycystic ovarian syndrome, obesity, gestational diabetes mellitus, and cancer [19]. Metformin has a relatively high allowed dosage up to 2–3 g/day in Europe and the USA [20]. Therefore, the limit for NDMA and other NAs has been set according to the maximum daily dosage (3 g/day). Acceptable intake of NDMA according to EMA is 92 ng/day (the acceptable intake is a daily exposure to a compound, such as NDMA, NDEA, or *N*-nitrosomethylbutylamine (NMBA), that approximates a 1:100,000 cancer risk after 70 years of exposure). Therefore, recommended the maximum allowed limit of NDMA in the metformin products to be set at 0.032 ppm.

The main aim of the present study was to assay the NDMA content in the metformin API and finished products. The gas chromatography-tandem mass spectrometry (GC-MS/MS) method for quantification of NDMA and seven additional NAs was developed and validated. Additional attempts that can explain the mechanism of NDMA formation during the manufacturing process were conducted. The developed method was successfully applied to the analysis of 105 different metformin products of 13 different companies providing detailed insight into various degrees of NDMA contamination in metformin finished products on the European market.

## 2. Results

### 2.1. Validation Results

**Figure 1:**
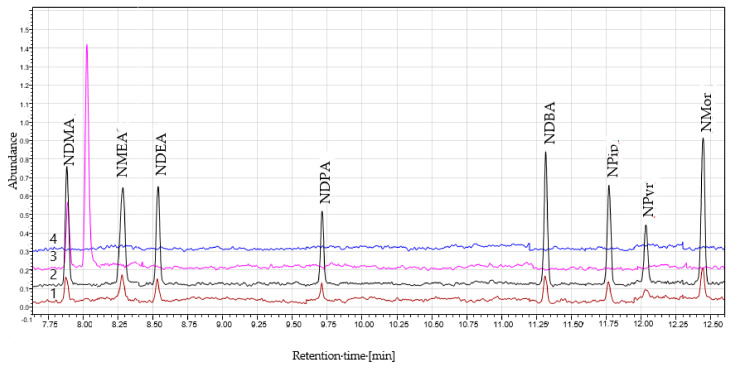
The chromatograms of (**1**) the N-nitrosamines (NAs) standards (1.0 ng/mL); (**2**) the NAs standards (5.0 ng/mL); (**3**) the sample solution; (**4**) the blank solution.

The chromatograms of the NAs standards, the sample solution, and the blank solution are presented in Figure 1. An additional peak at approximately 8 min was observed in the sample solution depending on the finished product manufacturer. The peak had a single MRM transition of the NDMA (77 → 44.10) and did not co-elute with NDMA. Additional studies revealed that the peak can be *N*,*N*-dimethylformamide (DMF), which can be used in the synthesis of metformin. Therefore, DMF appears to be a residual solvent from the synthesis.

Good linearity for each analyte can be achieved by using linear regression over the 0.5–9.5 ng/mL calibration range. The coefficients of determination of the calibration curves were ≥0.995 for all analytes. The results of the precision and accuracy estimates of the calibration standards for each analyte are summarized in Table 1. The obtained accuracy parameters were ranging from 92.61% to 105.03% for all analytes. The precision parameters for all NAs in all concentrations were below <15%, with a maximum achieved at 10.55%.

The triplicate analysis of the finished product yielded good precisions for NDMA assay ranging from 0.36% to 16.28%, thus verifying the repeatability of the assay applied to the finished products containing various concentrations of the corresponding analytes. Higher relative standard deviations were obtained in the prolonged dosage finished products (up to 22.09%), probably due to hindered extraction of the formed gel of carboxymethyl cellulose used in the tablet formulations.

**Figure 2:**
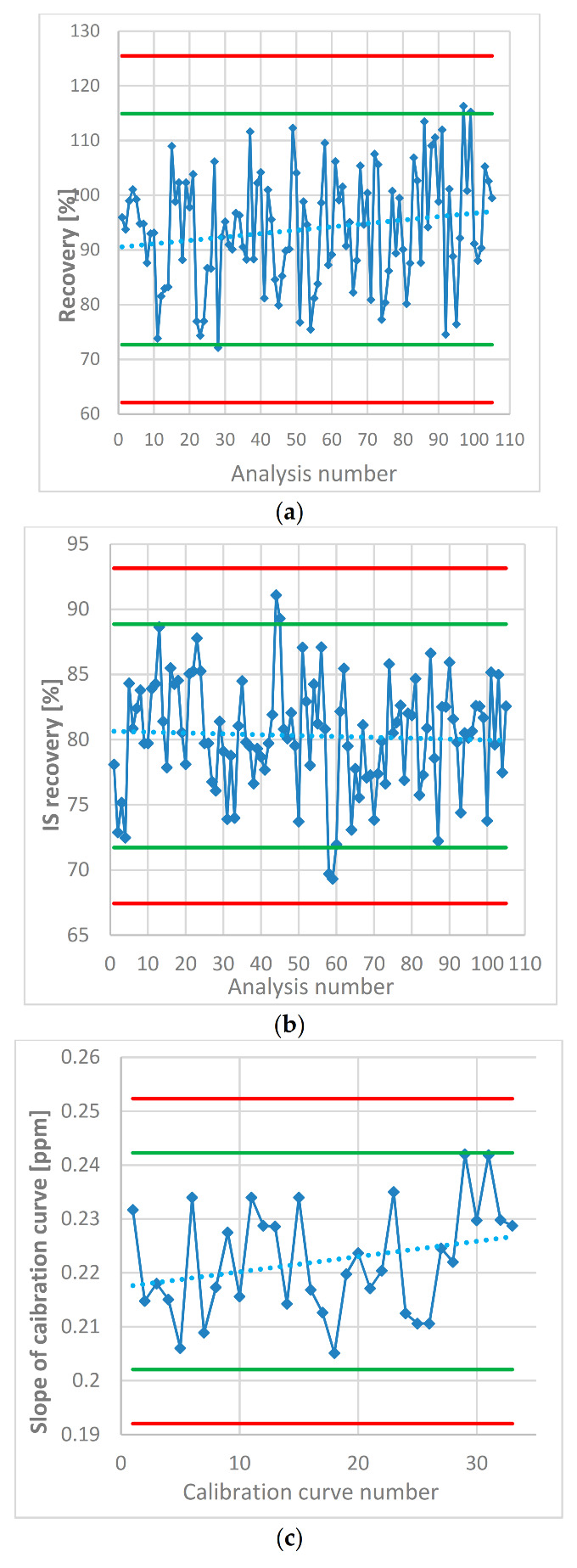
(**a**) NDMA recovery in the spiked samples estimated in each analysis of the metformin products. (**b**) Internal standard (IS – d_6_-NDMA) recovery as a percentage of the mean peak area in the standard solutions. (**c**) Slope of the calibration curves of NDMA calculated in each analysis. Green lines represent ± 2 × SD, red lines represent ±3 × SD.

The recovery of each spiked solution has been calculated. Figure 2 (1) presents the NDMA recoveries of the spiked samples. The calculated recovery was found to be outside of the ±2 × SD range of obtained mean recovery only three times of all tests performed however, the set limit for the recovery was 70–130%, meaning that each analysis remains valid. Additionally, the yield of the extraction of NDMA from water solution to CH_2_Cl_2_ was controlled by the IS − d_6_-NDMA recovery. According to Figure 2 (2), the recovery values were from 69.31 to 91.08%.

In each analysis, the NDMA content was determined using the standard curve. The concentration of the standards was the same in every analysis. Therefore, the parameters that describe the linearity can be compared through the whole study that included 105 samples. Figure 2 (3) shows the changes in one of the most important parameters, which influences the measurement in the majority of the samples, the slope of the calibration curves. The slope value appears to have a slight trend towards a higher value, which may be due to the evaporation of the stock standard solution (dissolved in MeOH) despite being stored in a freezer.

The main aim of the proposed GC-MS/MS method is the determination of NDMA in the metformin API or finished products. However, chromatography was developed to quantify all listed NAs. The standard and spike solutions were prepared only with the NAs mixture. The chromatography method was developed to evaluate each prepared sample and estimate whether it is contaminated by mistake at any point in the preparation. If the samples contain any additional NAs apart from NDMA, an investigation should be performed to determine if there was a mistake in the sample preparation or if metformin is indeed contaminated with another NA. However, such an event did not happen throughout the whole study of 105 samples, and no additional NAs were detected in the metformin products.

### 2.2. Artifact Formation

Possible artifact formation at any point during the sample preparation, and GC analysis is one of the most important issues of metformin analysis. It is worth mentioning that during the development of the method, we have experienced issues due to artifact formation in the sample solution. In the beginning, the sample preparation method was based on the direct extraction of the metformin API in the CH_2_Cl_2_ solution containing the IS. Various syringe filters were tested to remove undissolved metformin from the CH_2_Cl_2_ solution. Based on the acquired results, we concluded that an artifact was experienced when the nylon syringe filters were used. The amount of NDMA formation after the filtration differed based on the time of the filtration and the brand of the filter used. However, during the method development, there was no information about the NDMA origin/formation in metformin, and thus, NDMA could have been entrapped in the metformin crystals; based on the obtained results, development of the method for direct CH_2_Cl_2_ extraction was discontinued, and the extraction of the aqueous solution of metformin was pursued.

### 2.3. Results of the Assay of NDMA Concentration

**Figure 3:**
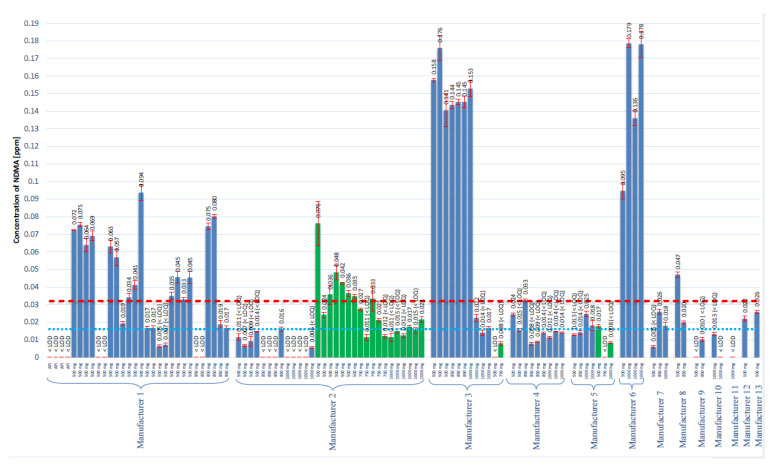
The results of the assay of NDMA concentration in all tested metformin products. Blue bars represent finished products, and the green bars corresponds to the prolonged finished products. The red dotted line represents the suggested maximum allowed concentration of 0.032 ppm. The blue dotted line represents the LOQ of the method that corresponds to 0.016 ppm.

All tested samples met the criteria of the System Suitability Test established in Section 4.5.4; therefore, it was concluded that all conducted tests were valid. NDMA was found in 86 (81.9%) of all 105 products tested, including 64 (85.3%) metformin finished products and 22 (91.7%) prolonged dosage finished products. The average content (range) in the 500, 850, and 1000 mg metformin finished products was 0.063 ppm (0.017–0.176 ppm), 0.065 ppm (0.016–0.145 ppm), and 0.083 ppm (0.017–0.179 ppm), respectively. The average content (range) in the 500, 750, and 1000 mg metformin prolonged dosage finished products was 0.038 ppm (0.017–0.076 ppm), 0.027 ppm (0.021–0.033 ppm), and 0.019 ppm (0.017–0.021 ppm), respectively. The results of the analyzed products are presented in Figure 3. Based on the recommendation of the EMA and on our data, 28 finished products and 7 prolonged dosage products were found to exceed the acceptable daily intake limit with regard to the NDMA contamination.

### 2.4. Study of Possible Formation Routes of NDMA

To ensure that NDMA does not originate from one of the excipients, the developed and validated method was used to analyze the additional substances. The excipients available for the testing included sodium carboxymethyl starch (type A), povidone (K30), colloidal silica (anhydrous), corn starch, and magnesium stearate. The recovery of NDMA in each substance was verified. The recovery in the excipient analysis was 87.75%, 108.50%, 98.52%, 80.76%, and 85.58%, respectively. Therefore, we concluded that the method can be used to control the excipients. However, no contamination with NDMA was detected in the analyzed samples.

**Figure 4:**
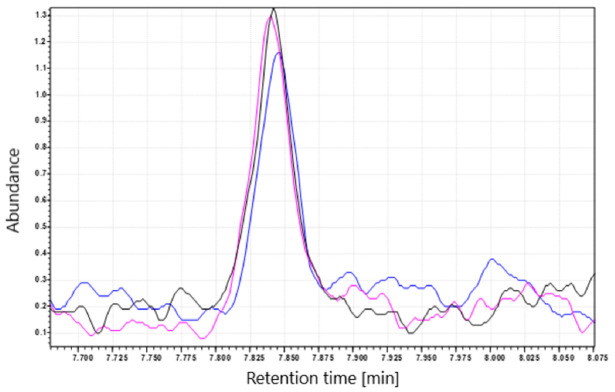
NDMA peak before and after treatment with a short period of high temperature (100 °C). The black line (–) corresponds to tablets extracted with CH_2_Cl_2_ (control), (pink line) tablets heated for 30 s; (blue line) tables heated for 2 min.

Additionally, to ensure that NDMA does not form during heating of the tablets in the blistering process, we subjected the metformin tablets to a short period (30 s and 2 min) of heating at high temperature (100 °C). After heating, the concentration of NDMA in the tablets was decreased (Figure 4). The decrease was observed in tablets containing NDMA below and above the recommended limit. The decrease in the determined NDMA concentration may be caused by NDMA evaporation from the surface of the tablets.

Heating the tablets containing the NDMA levels above and below the set limit (0.032 ppm) at high temperature (100 °C) did not increase NDMA concentration, and instead decreased it—probably as a result of NDMA evaporation.

## 3. Discussion

The gas chromatography and MRM transition method were optimized for eight common NAs. There are a number of available instrumental methods for NAs analysis, and the methods of the gas and liquid chromatography are the most popular choice.

The selection of a column for gas chromatography can include non-polar columns (e.g., 5% phenyl-methylpolysiloxane, HP-5MS and DB-5MS), intermediate polarity columns (e.g., 35% phenyl-methylpolysiloxane, HP-35MS) and high polarity columns (e.g., polyethylene glycol, DB-WAX and TG-WAX) [21]. Analysis of NAs requires very low detection limits, and therefore, the use of mass spectrometry, preferably with the MS/MS mode, is the obvious choice. The ionization sources previously used in the analysis of NAs include electron impact (EI) with MS/MS mode [22,23] and high-resolution mass spectrometry (HRMS) [24] with positive chemical ionization (PCI) using methanol [25], acetonitrile [26], ammonia [27], or methane [21] as the reagent gas. CI has been shown to yield better specificity than that of EI and CI, thus provides higher sensitivity in the NAs analysis [28]. However, EI remains the most popular choice for GC-MS/MS, due to its ability of simultaneous identification by comparing the registered spectra with the spectra stored in the mass spectra library. Alternatively, The NA analysis can be performed using liquid chromatography on a reversed-phase column (mainly C8 or C18) using a combination of electrospray ionization (ESI) or atmospheric pressure chemical ionization (APCI) as ionization method used in a mass spectrometer [13]. However, in addition to chromatography and mass spectrometry, sample preparation is an important part of NAs analysis; in the case of GC, the injection method is also important. Injection of the samples onto the GC system for NAs analysis can use classical split/splitless [29], programmed temperature or cold injector [30,31], thermal desorption (TD), or headspace (HS) injection [32]. Sample preparation can involve distillation, liquid-liquid extraction, and/or SPE. However, various pitfalls of complex sample preparation steps have been reported in the analysis of NAs in various matrices. The combination of liquid-liquid extraction with CH_2_Cl_2_, which is the most popular extraction solvent for NAs, with splitless injection in GC appears to be the most frequently used in the case of analysis of sartans by Official Medicines Control Laboratories (OMCLs) [13]. A similar solution was also already used in the method originated from Singapore; however, sample preparation requires using 0.1 M solution of hydrochloric acid as a water phase [33]. In order to minimize the risk of the unwanted reaction between hydrochloric acid or its impurities, it was decided to use deionized water instead. Nevertheless, using CH_2_Cl_2_ is apparently the easiest, the fastest, and robust sample preparation; all the properties are required in the quality control of the medicinal products.

In addition to the studies of NDMA contamination in the metformin finished products, experiments were conducted to further investigate the NA formation mechanism. The most obvious way to prove that NDMA originates during the manufacturing process is to measure NDMA in API and in finished products in which this particular API was used. Figure 3 represents the results (Manufacturer 1), obtained with four different batches of API and 24 different batches of finished products manufactured with analyzed API. The content of NDMA in API was always below the LOD; however, in the finished product, NDMA presence was detected in 22 batches, and 15 batches had NDMA level in excess of the 0.032 ppm recommended limit. Therefore, the manufacturing process of various batches of the excipients has an impact on the NDMA content. We received a list of all excipients used in the manufacturing process from Manufacturer 1 to evaluate possible NDMA contamination. However, no NDMA was found in all provided substances, and we, thus, conclude that the excipients are not the source of NDMA.

Another result confirming that NDMA is manufacturing contamination that was obtained in the batches from Manufacturers 2 and 3. The problem apparently occurs in the manufacturing process of prolonged dosage products. The manufacturing process and the excipients were different from those in the finished product. A similar situation occurs in the case of Manufacturer 3; seven batches had NDMA concentration above 0.140 ppm, and five batches had NDMA concentrations below 0.032 ppm (two batches with prolonged dosage). After these results were obtained, we have identified that these five and seven batches were manufactured in different places. Therefore, the manufacturing line and excipients were probably different, and as a result, the NDMA content was also different.

One of the hypotheses on the mechanism of the NDMA formation during the manufacturing process is that dimethylamine (DMA, impurity F, according to European Pharmacopeia, 0,05% *w/w* allowed) present in the metformin API is reacting with the residual nitrites/nitrates from the excipients during the wet granulation process. However, heating of the API containing 0.03% DMA mixed with sodium nitrite and sodium nitrate did not generate NDMA-positive results (data not shown). Despite that heating metformin API with nitrites/nitrates, it is not an actual manufacturing process, some other factor probably is needed to catalyze the NDMA formation, and this factor is delivered through one of the excipients; at this point, the factor eludes detection.

Another possible hypothesis is that NDMA is formed during the blistering process of the finished product (the tablets in blisters are exposed to transient high temperature during film sealing) or is present as an impurity of the ink, which is used to print on the aluminum foil. However, our study indicates that NDMA is not a surface impurity, but is rather an impurity that is present in the whole tablet mass. Heating the tablets at a high temperature (100 °C) with NDMA levels above or below the set limit (0.032 ppm) does not increase NDMA concentration, and instead decreases it—probably as a result of NDMA evaporation. However, the is a possibility that during the high-temperature treatment, the substrates needed to form NDMA have already reacted, and heating the tablets for the second time does not cause additional NDMA formation. Additionally, we received a batch from one of the manufacturers sampled before the blistering process (bulk batch); we have assayed the NDMA concentration in this batch, and it was above the recommended limit of 0.032 ppm. Therefore, we conclude that increased temperature present during the blistering process is not responsible for the NDMA formation.

## 4. Materials and Methods

### 4.1. Materials

All solvents for analysis were of MS-grade quality. Dichloromethane (CH_2_Cl_2_) and methanol (MeOH) were purchased from Merck Millipore (Germany, Darmstadt). The NAs mix in MeOH was purchased from Sigma-Aldrich (Poznań, Poland), which contained NDMA, NMEA, NDEA, NDPA, NDBA, NPip, NPyr, and NMor in a concentration of 2 mg/mL of each component. The deuterated d_6_-NDMA (99.95 atom % D; 1 mg/mL) in CH_2_Cl_2_ was obtained from LGC Standards (Łomianki, Poland). Finished medicinal products were acquired from several marketing authorizations holders (MAHs). The API and excipients (sodium carboxymethyl starch (type A), povidone (K30), colloidal silica (anhydrous), corn starch, and magnesium stearate) available for the testing were delivered by one of the manufacturers.

### 4.2. GC-MS Method of Nitrosamine Determination

A Shimadzu GC-2010 Plus system with an Optic-4 autosampler coupled with an MS-TQ8050 mass spectrometer was used. NAs were separated on a TG-WAXMS column (30 m × 0.25 mm, film thickness 0.5 μm). The carrier gas was helium with a flow rate of 36.2 cm/sec (1 mL/min). The initial column temperature of 45 °C was held for 3 min and then programmed to increase at 25 °C/min to 130 °C, at 12 °C/min to 200 °C, and at 20 °C/min to 250 °C, where it was held for 3 min. Injections (2.0 µL) were made in the splitless program mode. Injector temperature was 250 °C with a high-pressure injection (300 kPa) for 1 min. The transfer line temperature was 250 °C, ion source temperature was 250 °C, and the energy of the electron impact ionization was 70 eV. The method parameters are listed in Table 2. NAs were analyzed using the MRM transition listed in Table 3.

### 4.3. Standard Solution Preparation

The internal standard (IS) solution (100 ng/mL) was prepared from the commercially available d_6_-NDMA standard (1 mg/mL) and diluted with CH_2_Cl_2_. The stock standard solution (1 µg/mL) was prepared from the available standard of NAs in a 50 mL volumetric flask in MeOH. Then, the solution was diluted to 10 ng/mL with CH_2_Cl_2_. The calibration curve was prepared at concentrations of 0.5; 1.0; 1.5; 2.0; 2.5; 5.0; 7.5; and 9.5 ng/mL by addition of the internal standard to each solution to the final concentration of 5 ng/mL d_6_-NDMA. The spiking solution was prepared by diluting the prepared NAs solution in MeOH to 10 ng/mL with deionized water.

### 4.4. Sample and Spiked Sample Preparation

#### 4.4.1. Active Pharmaceutical Ingredient (API)

For API samples, 500 mg of the active ingredient was weighed into a 15 mL centrifuge tube. Metformin was dissolved in 7 mL deionized water; 400 µL of IS solution was added, briefly vortexed, and then shaken well for at least 5 min. CH_2_Cl_2_ (7.6 mL) was added to the solution, briefly vortexed, and then shaken well for at least 10 min. The suspension was then centrifuged at approximately 10,000× *g* for at least 10 min. The lower organic phase was transferred to a clean sample vial. The extraction process was prepared three times from independent weights.

For the spiking test solution, the same amount of API was weighed into a 15 mL centrifuge tube; 1.6 mL of the spiking solution was added along with 5.4 mL water, and the tube was mixed and extracted with CH_2_Cl_2_ as described above.

#### 4.4.2. Finished Product

A representative sample was prepared from approximately 10 ground tablets. The mixed tablet powder was weighed into a 15 mL centrifuge tube; the weight of the material corresponded to the amount of active ingredient of approx. Five hundred milligram and specific attention was paid not to exceed 1.5 g of total weight. The extraction process was prepared three times from independent weights. The extract and spiked solution were prepared, as described above for the API solution.

#### 4.4.3. Prolonged Dosage Finished Product

A representative sample was prepared from approximately 10 ground tablets. Mixed tablet powder was weighed into a 15 mL centrifuge tube; the weight of the material corresponded to the amount of the active ingredient of approx. 500 mg; specific attention was paid not to exceed 1.5 g of total weight.

The sample powder was mixed with 7 mL water, immediately mixed, and vortexed. Then, the sample was incubated for at least 10 min to dissolve the whole tablet mass in water. Then, 400 µL of IS solution and 3.6 mL of CH_2_Cl_2_ were added; the mixture was briefly vortexed, and shaken well to disperse the formed gel in the organic phase. CH_2_Cl_2_ (4 mL) was added to this suspension, briefly vortexed and then shaken well for at least 10 min. The suspension was then centrifuged at approximately 10,000× *g* for at least 10 min. The lower organic phase was transferred to a clean sample vial. The extraction process was prepared three times from independent weights. To prepare the spiking test solution, the same amount of the tablet mass was weighed into a 15 mL centrifuge tube; 1.6 mL of the spiking solution was added with 5.4 mL water; the solution was mixed, and extracted with CH_2_Cl_2_ as described above.

#### 4.4.4. Excipient Samples

To prepare the excipient samples, 1000 mg of an excipient (500 mg in case of colloidal silica) was weighed into a 15 mL centrifuge tube. The substances were dissolved or suspended in 7 mL deionized water; 400 µL of IS solution was added, vortexed briefly, and then shaken well for at least 5 min. CH_2_Cl_2_ (7.6 mL) was added to the solution, vortexed briefly, and shaken well for at least 10 min. The suspension was then centrifuged at approximately 10,000× *g* for at least 10 min. The lower organic phase was transferred to clean sample vial. The test samples were prepared from three independent weights. For the spiking test solution, the same amount of the substances was weighed into a 15 mL centrifuge tube; 1.6 mL of spiking solution was added with 5.4 mL water; the solution was mixed and extracted with CH_2_Cl_2_, as described above.

#### 4.4.5. Sample Blank Solution Preparation

The sample blank solution was prepared as described in Section 4.4.1, but without the addition of the sample.

### 4.5. Validation of the GC-MS/MS Method

#### 4.5.1. Specificity

The specificity of the method of NA assay was verified by comparing the chromatograms of the contaminant-free samples (finished and prolonged dosage form products) before and after spiking with the corresponding analytes. No peaks from the tablet matrix should coelute at the retention times of any of the NAs.

#### 4.5.2. Linearity, Precision and Accuracy, Limits of Detection (LOD) and Limits of Quantification (LOQ)

Analytes were quantified using d_6_-NDMA as IS for all NAs. For the determination of linearity of the NDMA, NDEA, NDPA, NDBA NPip, Npyr, and Nmor assay three eight-point calibration curves were constructed as described in Section 4.3. The calibration curves were evaluated individually by linear regression. The slopes, intercepts, and the coefficients of determination of the corresponding individual curves were calculated. For acceptable linearity, the coefficient of determination of each calibration curve should be R^2^ ≥ 0.995.

The acceptable accuracy of six injections of three different concentration, including 1.5 ng/mL, 2.0 ng/mL, and 2.5 ng/mL, expressed as the percentage of the measured concentration versus the theoretical concentration of the prepared solutions, should be in a range between 90–110%. The precision, expressed as the relative standard deviation, should not exceed 15% at each concentration level tested.

LODs and LOQs were calculated using the linearity curves based on the standard deviation of the response (σ) and the slope of the curves (S) using the multiplier suggested by the ICH standard. The LOD and LOQ were calculated from the following equations: LOD = (3.3 × σ/S) and LOD = (10 × σ/S).

#### 4.5.3. Recovery

In each metformin sample analysis, the spike solution was prepared by spiking NAs solution to an obtained a final concentration of 2.0 ng/mL NAs in the organic phase. The recovery was calculated as the ratio of subtracted mean results of the sample from the calculated result of the spiked solution, to the theoretical spiking concentration. Additionally, the recovery of the IS was calculated as a ratio of the mean IS peak area in the sample solution to the mean IS peak area in the standard solution.

#### 4.5.4. System Suitability Test

To validate each test, the listed conditions have to be met: No interfering signals in the sample blank solution; no significant co-elution in the spiked sample solutions; S/N of the spiked test solutions has to be at least 10 for all spiked NAs signals; correlation of the calibration curves has to be at least 0.995; area of d_6_-NDMA in the sample solution has to be at least 60% of the mean area of d_6_-NDMA in the standard solutions; and recovery in the spiked sample solution has to be between 70% and 130%.

## 5. Conclusions

After the discovery of the presence of NAs in sartans and ranitidine (especially NDMA and NDEA), the metformin finished products are other medications that have been found to be contaminated with NDMA. After the sartans and ranitidine event, the scientists should have been more prepared for this type of crisis. However, the reality seems to be as brutal as always. Resolving the unexpected events in the case of sartans and ranitidine was achieved by withdrawing the finished product from the market. On the other hand, the metformin finished products do not have any substitutes, which have a similar profile of action. The available substitutes for metformin are sulfonylurea derivatives (which possess a substantially higher possibility of adverse reactions) and insulin (which is a pro-injection drug that requires control of blood sugar and estimation of the dose by the patient). Therefore, a similar solution is not applicable in this case.

The GC–MS/MS method developed for the quantitation of eight NAs, including NDMA, NMEA, NDEA, NDPA, NDBA, NPip, NPyr, and NMor, was found to be suitable for the determination of traces of the analytes according to the validation results. Almost one-third of all tested samples were found to contain NDMA levels above the recommended limit based on maximum daily dosage. To the best of our knowledge, this is the first report that describes the measured concentrations of NDMA in the metformin medicinal products. The obtained results show the actual seriousness of the situation. Therefore, manufacturers, regulatory offices, and OMCLs need to cooperate to solve this problem. In our opinion, the NDMA contamination in the metformin finished products should be considered as an unforeseen event, which was not influenced by pharmaceutical companies/manufacturers; therefore, it should not jeopardize the reputation of a pharmaceutical company at any point. However, the problem of the formation of NDMA during the manufacturing process remains unresolved; hence, routine analysis of the finished products has to be applied to guarantee the quality and safety of available medications.

## Figures and Tables

**Table 1:** Validation parameters for GC-MS/MS analysis of NAs.

NA	Linearity Range	Equation	R^2^ (*n* = 8)	LOD	LOQ	Final Concentration after NA Addition	Accuracy	Precision
				[ng/mL]	[ng/mL]			
*N*-nitrosodimethylamine **NDMA**	0.5–9.5 ng/mL	y = 0.216x − 0.001	0.9990	0.33	1.00	1.5 ng/mL	99.60%	6.01%
						2.0 ng/mL	99.52%	3.42%
						2.5 ng/mL	100.21%	4.85%
*N*-nitrosomethylethylamine **NMEA**		y = 0.301x − 0.068	0.9997	0.16	0.49	1.5 ng/mL	105.03%	4.76%
						2.0 ng/mL	104.08%	5.21%
						2.5 ng/mL	100.03%	5.48%
*N*-nitrosodiethylamine **NDEA**		y = 0.203x − 0.023	0.9970	0.32	0.97	1.5 ng/mL	104.11%	6.27%
						2.0 ng/mL	106.13%	3.17%
						2.5 ng/mL	103.69%	5.58%
*N*-nitrosodi-n-propylamine **NDPA**		y = 0.145x − 0.014	0.9992	0.26	0.79	1.5 ng/mL	98.99%	6.91%
						2.0 ng/mL	101.88%	3.23%
						2.5 ng/mL	103.09%	5.92%
*N*-nitrosodi-n-butylamine **NDBA**		y = 0.257x − 0.040	0.9960	0.48	1.46	1.5 ng/mL	92.61%	8.10%
						2.0 ng/mL	96.31%	6.79%
						2.5 ng/mL	95.17%	2.50%
*N*-nitrosopiperidine **NPip**		y = 0.172x + 0.002	0.9976	0.45	1.36	1.5 ng/mL	98.39%	6.79%
						2.0 ng/mL	102.99%	8.89%
						2.5 ng/mL	99.04%	4.73%
*N*-nitrosopyrrolidine **NPyr**		y = 0.124x − 0.013	0.9958	0.49	1.48	1.5 ng/mL	97.53%	7.56%
						2.0 ng/mL	103.05%	5.53%
						2.5 ng/mL	103.22%	5.94%
*N*-nitrosomorpholine **NMor**		y = 0.247x − 0.023	0.9981	0.39	1.20	1.5 ng/mL	101.76%	10.55%
						2.0 ng/mL	100.18%	8.07%
						2.5 ng/mL	98.96%	5.78%

**Table 2:** Parameters of the developed GC-MS method.

**GC-Parameters**	
Column	TG-WAXMS 30 m × 0.25 mm; 0.5 µm
Carrier gas	Helium
Flow rate	36.2 cm/s (1 mL/min)
Injector port temp.	250 °C
Injection volume	2 µL
Injection mode	Splitless, high-pressure injection 300 kPa
Oven program	45 °C hold time 3 min 130 °C rate 25 °C hold time 0 min 200 °C rate 12 °C hold time 0 min 250 °C rate 20 °C hold time 3 min
**MS-Parameters**	
Ion Source Temperature	250 °C
Interface Temperature	250 °C
Fixed Electron Energy	70 eV
Acquisition Type	MRM
Solvent Delay	5 min
Collision Gas	argon 200 kPa

**Table 3:** MRM transitions for analyzed NAs.

Component	Quantitative Transition			Qualitative Transition		
	Precursor Ion (*m/z*)	Product Ion (*m/z*)	CE (V)	Precursor Ion (*m/z*)	Product Ion (*m/z*)	CE (V)
NDMA	74	44.10	5	74	42.10	14
d_6_-NDMA	80	50.10	5	80	46.10	14
NMEA	88	71.10	5	88	73.10	7
NDEA	102	85.10	5	102	56.10	14
NDPA	130	113.20	5	130	88.10	9
NDBA	116	99.10	5	158.15	99.10	7
Npip	114	84.10	8	114	97.10	8
Npyr	100	55.10	7	100	68.10	15
Nmor	116	86.10	3	116	56.10	23

## References

[1] Snodin D.J., Elder D.P. (2019). Short commentary on NDMA (*N*-nitrosodimethylamine) contamination of valsartan products. Regul. Toxicol. Pharmacol..

[2] Shen R., Andrews S.A. (2013). NDMA formation from amine-based pharmaceuticals—Impact from prechlorination and water matrix. Water Res..

[3] Brendler S.Y., Tompa A., Hutter K.F., Preussmann R., Pool-Zobel B.L. (1992). In vivo and in vitro genotoxicity of several N-nitrosamines in extrahepatic tissues of the rat. Carcinogenesis.

[4] Wang H.-Y., Qin M., Dong L., Lv J.-Y., Wang X. (2017). Genotoxicity of a Low-Dose Nitrosamine Mixture as Drinking Water Disinfection Byproducts in NIH3T3 Cells. Int. J. Med. Sci..

[5] La Vecchia C., D’avanzo B., Airoldi L., Braga C., DeCarli A. (1995). Nitrosamine intake and gastric cancer risk. Eur. J. Cancer Prev..

[6] Knekt P., Dich J., Hakulinen T. (1999). Risk of colorectal and other gastro-intestinal cancers after exposure to nitrate, nitrite and N-nitroso compounds: A follow-up study. Int. J. Cancer.

[7] Pottegård A., Kristensen K.B., Ernst M.T., Johansen N.B., Quartarolo P., Hallas J. (2018). Use of N-nitrosodimethylamine (NDMA) contaminated valsartan products and risk of cancer: Danish nationwide cohort study. BMJ.

[8] World Health Organization (1993). Guidelines for Drinking-Water Quality.

[9] United States Environmental Protection Agency *Technical Fact Sheet—N-Nitroso-dimethylamine (NDMA)*; 2014. https://www.epa.gov/sites/production/files/2014-03/documents/ffrrofactsheet_contaminant_ndma_january2014_final.pdf.

[10] Crews C. (2010). The determination of N-nitrosamines in food. Qual. Assur. Saf. Crop. Foods.

[11] Herrmann S.S., Duedahl-Olesen L., Granby K. (2015). Occurrence of volatile and non-volatile N-nitrosamines in processed meat products and the role of heat treatment. Food Control..

[12] Buckingham L. (2020). Suspension of Ranitidine Medicines in the EU. https://www.ema.europa.eu/en/news/suspension-ranitidine-medicines-eu.

[13] Sörgel P.F., Kinzig M., Abdel-Tawab M., Bidmon C., Schreiber A., Ermel S., Wohlfart J., Besa A., Scherf-Clavel O., Holzgrabe U. (2019). The contamination of valsartan and other sartans, part 1: New findings. J. Pharm. Biomed. Anal..

[14] Parr M., Joseph J.F. (2019). NDMA impurity in valsartan and other pharmaceutical products: Analytical methods for the determination of N-nitrosamines. J. Pharm. Biomed. Anal..

[15] Munch J.W., Bassett M.V. (2004). Determination of Nitrosamines in Drinking Water by Solid Phase Extraction and Capillary Column Gas Chromatography with Large Volume Injection and Chemical Ionization Tandem Mass Spectrometry (MS/MS). https://cfpub.epa.gov/si/si_public_record_report.cfm?Lab=NERL&dirEntryId=103912.

[16] Common Heart Drug Recalled in 22 Countries for Possible Cancer Link—CNN. https://edition.cnn.com/2018/07/06/health/valsartan-heart-drug-recall-intl/index.html.

[17] Francisco E.M. (2019). EMA Update on Metformin Diabetes Medicines. https://www.ema.europa.eu/en/news/ema-update-metformin-diabetes-medicines.

[18] Rojas L.B.A., Gomes M.B. (2013). Metformin: An old but still the best treatment for type 2 diabetes. Diabetol. Metab. Syndr..

[19] Nasri H., Rafieian-Kopaei M. (2014). Metformin: Current knowledge. J. Res. Med. Sci..

[20] Odawara M., Kawamori R., Tajima N., Iwamoto Y., Kageyama S., Yodo Y., Ueki F., Hotta N. (2017). Long-term treatment study of global standard dose metformin in Japanese patients with type 2 diabetes mellitus. Diabetol. Int..

[21] Sieira B.J., Carpinteiro I., Rodil R., Quintana J.B., Cela R. (2020). Determination of N-Nitrosamines by Gas Chromatography Coupled to Quadrupole–Time-of-Flight Mass Spectrometry in Water Samples. Separations.

[22] Lim H.-H., Oh Y.-S., Shin H.-S. (2020). Determination of N-nitrosodimethylamine and N-nitrosomethylethylamine in drug substances and products of sartans, metformin and ranitidine by precipitation and solid phase extraction and gas chromatography–tandem mass spectrometry. J. Pharm. Biomed. Anal..

[23] Yahaya A. (2019). Method development for the identification and quantitative analysis of seven nitrosamines using gas chromatography mass spectrometry. Chem. Data Collect..

[24] Planas C., Palacios O., Ventura F., Rivera J., Caixach J. (2008). Analysis of nitrosamines in water by automated SPE and isotope dilution GC/HRMSOccurrence in the different steps of a drinking water treatment plant, and in chlorinated samples from a reservoir and a sewage treatment plant effluent. Talanta.

[25] Llop A., Borrull F., Pocurull E. (2012). Pressurised hot water extraction followed by headspace solid-phase microextraction and gas chromatography–tandem mass spectrometry for the determination of N-nitrosamines in sewage sludge. Talanta.

[26] Munch J.W., Bassett M.V. (2006). Method development for the analysis of N-nitrosodimethylamine and other N-nitrosamines in drinking water at low nanogram/liter concentrations using solid-phase extraction and gas chromatography with chemical ionization tandem mass spectrometry. J. AOAC Int..

[27] Sannino A., Bolzoni L. (2013). GC/CI–MS/MS method for the identification and quantification of volatile N-nitrosamines in meat products. Food Chem..

[28] Yoon S., Nakada N., Tanaka H. (2012). A new method for quantifying N-nitrosamines in wastewater samples by gas chromatography—Triple quadrupole mass spectrometry. Talanta.

[29] Ramezani H., Hosseini H., Kamankesh M., Ghasemzadeh-Mohammadi V., Mohammadi A. (2014). Rapid determination of nitrosamines in sausage and salami using microwave-assisted extraction and dispersive liquid–liquid microextraction followed by gas chromatography–mass spectrometry. Eur. Food Res. Technol..

[30] Shen R., Andrews S.A. (2011). Demonstration of 20 pharmaceuticals and personal care products (PPCPs) as nitrosamine precursors during chloramine disinfection. Water Res..

[31] Empl M.T., Kammeyer P., Ulrich R., Joseph J.F., Parr M., Willenberg I., Schebb N.H., Baumgärtner W., Röhrdanz E., Steffen C. (2014). The influence of chronic l-carnitine supplementation on the formation of preneoplastic and atherosclerotic lesions in the colon and aorta of male F344 rats. Arch. Toxicol..

[32] US Food and Drug Administration (2019). GC/MS Headspace Method for Detection of NDMA in Valsartan Drug Substance and Drug Products. https://www.fda.gov/drugs/drug-safety-and-availability.

[33] Update on Impurities in Metformin Products. https://www.hsa.gov.sg/announcements/safety-alert/update-on-impurities-in-metformin-products.

